# Bridging the Communication Gap Between People With Cognitive Impairments and Their Caregivers Using mHealth Apps: User-Centered Design and Evaluation Study With People With 22q11 Deletion Syndrome

**DOI:** 10.2196/44290

**Published:** 2023-08-16

**Authors:** Martijn Van Dooren, Robin De Croon, Ann Swillen, Katrien Verbert

**Affiliations:** 1 Department of Computer Science KU Leuven Leuven Belgium; 2 Department of Human Genetics KU Leuven Leuven Belgium; 3 Center for Human Genetics University Hospital Gasthuisberg Leuven Leuven Belgium

**Keywords:** 22q11 deletion syndrome, 22q11 DS, cognitive impairments, communication gap, mHealth

## Abstract

**Background:**

In families with children with cognitive impairments, both parents and children experience tension and have questions because of a lack of communication and adequate information. Therefore, there is a great need to develop tools that can help bridge the communication gap between patients and caregivers by stimulating conversations and providing psychoeducational tools. mHealth apps show great potential in this context.

**Objective:**

The objective of this research is to discover the specific ways young people with cognitive impairments and their families interact with mHealth apps in the context of bridging the communication gap. This newly discovered information leads to potentially more impactful mHealth interventions in the future. Therefore, this paper documents the design and development of a mHealth app for a specific group of people with cognitive impairments—people with 22q11 deletion syndrome (22q11 DS)—and their caregivers, as well as key learnings from the evaluation of this app.

**Methods:**

An iterative, user-centered design approach is used to design and develop the app. Design and evaluation happens in 2 phases. During the design phase, feedback is gathered from 2 medical experts and 3 human computer interaction (HCI) experts using a low-fidelity paper prototype. During the evaluation phase, feedback is gathered from 8 families with a child with 22q11 DS using a fully working proof of concept. This phase consists of a semistructured interview, a 2-4–week trial period, and a concluding semistructured interview.

**Results:**

The evaluation results of the fully working proof of concept led to design recommendations related to four different topics: (1) overcoming usage barriers, (2) stimulating conversation through a mHealth app, (3) providing information, and (4) bringing continual added value. Results are presented according to six different categories obtained in a thematic analysis: (1) feedback about the app “as is,” (2) difficulties, (3) comparison between physical and digital tool, (4) extensions, (5) intention, and (6) other.

**Conclusions:**

In this research, the need for apps that help bridge the communication gap between a person with cognitive impairment and their caregiver is confirmed. All participating families express their gratitude and mention the added value for other families. Therefore, it is highly encouraged for clinics and institutions to take action and develop an app to be used in practice. Furthermore, considerations when developing for people with 22q11 DS, or more broadly, people with cognitive impairments, are proposed. First, one should keep design principles in mind to overcome usage barriers. Next, recognition is a key concept when stimulating conversations through mobile apps. Third, information should be provided by a trusted source, and more than just clinical information can be considered valuable. Finally, having the possibility of using a digital tool that can be personalized brings continual added value.

## Introduction

### The Need for Bridging the Gap

During the last few decades, awareness and recognition about different genetic syndromes and genomic disorders have significantly increased. Specifically, for both 22q11 deletion syndrome (22q11 DS) and autism spectrum disorder, this has led to increased prevalence rates [1-4]. For Down syndrome, prevalence rates have been rising as well due to an increase in average maternal age [5,6]. People with both 22q11 DS and Down syndrome or autism spectrum disorder all have a high probability of having social and cognitive impairments [7-10]. Therefore, the increase in attention, awareness, and prevalence of these syndromes and disorders has led to increased attention for the needs of people with social cognitive impairments.

One subgroup of people with cognitive impairments will be the target group of this paper: young people with 22q11 DS. 22q11 DS is a congenital syndrome caused by a deletion or duplication on the long arm of chromosome 22 [11]. The prevalence rate of 22q11 DS is about 1 in 4500 [12], which makes it a rarer genetic syndrome than Down syndrome, which has a prevalence rate of about 1 in 720 [6]. People with 22q11 DS often have several social cognitive impairments, of which impaired emotion processing, circumscribed interests, deficits in sharing attention, gestural communication, initiating and maintaining conversations, and poor adaptive socialization are some examples [13,14].

Besides this, research states that “individuals with 22q11 DS seem to be aware of their health and psychological problems, but on the questions about social relationships and environment, they (possibly) respond with socially desirable answers. Individuals with 22q11 DS often want to please other people and do their very best in any circumstances. It is possible that they don’t want to bother anyone with their difficulties in social relationships and interaction with their environment” [15]. Combining all this, it can be concluded that it is not easy to have meaningful conversations about feelings, experiences, and symptoms with children with 22q11 DS. The lack of adapted and adequate communication can lead to frustration between people with 22q11 DS and their caregivers.

Bridging the communication gap between people with cognitive impairments and their caregivers is therefore a challenge present in many families today. The rise of modern technology potentially holds solutions to this challenge. mHealth apps could potentially be a great tool for supporting communication in these situations.

### mHealth Apps: a Viable Solution

In recent years, research has been done in the context of both mHealth apps for people with cognitive impairments and bridging the communication gap between people with cognitive impairments and their caregivers. First, literature indicates that families of patients with Down syndrome, Williams syndrome, and 22q11 DS showed a positive attitude toward mHealth technologies [16]. Besides, parents of children with 22q11 DS indicated they could have benefited from additional support to increase their confidence and success while disclosing the diagnosis to their child. Also, it could have increased the child’s comprehension of the information [17]. Combining these arguments, it might be valuable to investigate using mHealth apps with the specific goal of supporting communication for people with 22q11 DS and their caregivers.

Besides this observation, other mHealth apps that support families for other target groups were shown to have a high possibility of successful outcomes. In a family adaptation program for children with Down syndrome, all parents indicated they were willing to recommend this form of intervention to other families [18]. When using an mHealth resource for caregivers of cancer patients, these caregivers found the app highly useful in their experience of caregiving [19]. Lastly, in a scoping review to inform the development of mHealth apps for families with a child with Down syndrome, it was concluded that effective care coordination through such an app has the potential to increase family satisfaction [20].

Previous research also shows that developing mHealth resources specifically for people with 22q11 DS and other cognitive impairments has a great chance of helping them succeed in their goals. First, a remote cognitive remediation program with 22q11 DS youth was implemented without any problems [21]. This highlights the feasibility of any form of remote intervention for people with 22q11 DS. Furthermore, people with Down syndrome showed there are no hurdles to using any sort of touch gesture on a touchscreen [22,23]. As people with 22q11 DS likely have the same or better motoric abilities, this is an essential argument for the viability of an mHealth resource to support people with 22q11 DS.

Next, design implications were proposed to increase the potential success of mHealth apps for people with cognitive impairments. While designing these kinds of apps, keeping it simple, using visual cues, avoiding complex login functionality, using personalization, keeping patients’ mental models in mind, and employing a dynamic difficulty level are essential things to consider [24-26].

Combining all this, little research has been done on developing mobile mHealth apps for people with cognitive impairments and their caregivers in the context of bridging the communication gap between these 2. When considering people with 22q11 DS specifically, the need for research into these topics is even greater, as almost no research has been done concerning these matters. However, as all necessary building blocks are readily available, this study will focus on developing an mHealth app for people with 22q11 DS and their caregivers to help bridge the communication gap.

### Study Objective

The objective of this study is to gain further insight into how to build successful apps that support people with cognitive impairments and their families in the experience of caregiving. This study will focus specifically on people with 22q11 DS. The following research questions arise:

- Question 1: how can an mHealth app lower the communication burden between people with 22q11 DS and their caregivers (family and close friends)?
- Question 2: how can an mHealth app be a stimulant for people with 22q11 DS and their caregivers to have more regular conversations about the syndrome?
- Question 3: how can clinical information about the syndrome and the clinical symptoms of the condition be presented to young people with 22q11 DS and their caregivers to enhance health literacy?
- Question 4: which are the most important design principles when developing an app for young people with 22q11 DS?

A fully working proof of concept is designed, developed, and evaluated with young people with 22q11 DS and their families to formulate an answer to these research questions. The work in this paper contains valuable contributions in 2 areas. First, important design principles when designing for people with cognitive impairments, more specifically people with 22q11 DS, contribute to the health care informatics domain when considering mHealth apps for people with cognitive impairments. Besides this, important contributions are made to the psychoeducational domain by providing further insight into how to maximize the potential of a digital tool like the one created in this research.

## Methods

In the next parts, the full methodology used in this research is explained, referring to the overall study design, the participants in the research, the way data is analyzed, and the ethics approval granted.

### Study Design

**Figure 1:**
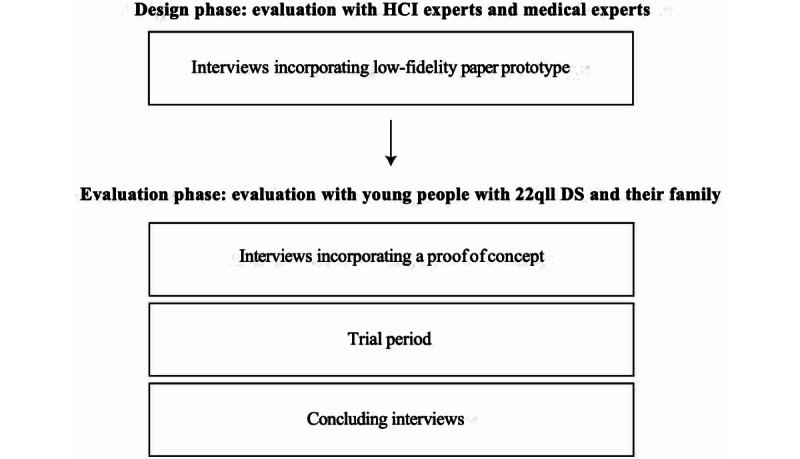
Visual overview of the study design.

This research incorporates an iterative, user-centered design process. By dividing the design process into different phases, insightful feedback from both experts and users is gathered. This research is split up into 2 main phases: a design phase that incorporates a low-level prototype and an evaluation phase that incorporates a fully working proof of concept. The latter again consists of 3 different parts: an initial interview that incorporates a first version of the proof of concept, a trial period, and a concluding interview. A visual overview of this study design can be found in Figure 1.

As a starting point for this research, a physical tool that was developed to support families with children with rare genetic syndromes is used as a starting point. The physical tool “Together we put the puzzle” launched in March 2020 [27]. The tool is currently regularly used in genetic counseling, mainly by clinical orthopedists, when parents and children need psychoeducation about their syndrome. Besides this, 50 families use the tool at home, and about 30 early intervention services, clinical genetic centers, special education schools, and rehabilitation centers are currently working with the psychoeducational tool (puzzle and booklet). Since April 2022, the tool has also been available in English, and 50 different copies have been sent to medical doctors (clinical geneticists, neuropediatricians, psychiatrists, etc) and allied health professionals (psychologists, remedial therapists, etc) working in the field of neurodevelopmental disorders due to a copy number variant (NDD-CNVs) across Europe.

#### Design Phase: Evaluation With Medical Experts and Human-Computer Interaction Experts Using a Low-Fidelity Paper Prototype

Using the concepts upon which the physical tool “Together we put the puzzle” is based, 2 prototypes are developed on paper. These low-fidelity prototypes are evaluated with both medical experts who have experience in the treatment of children with 22q11 DS and use “Together we put the puzzle” in practice and with experts in the human-computer interaction (HCI) domain. The latter was done to gain insight into the most prominent usability issues. The feedback was gathered in a web-based one-to-one think-aloud session of 45 minutes in which the 2 prototypes were shown to the users.

#### Evaluation Phase: User Evaluation With a Fully Working Proof of Concept

The second phase consists of 3 different parts. By conducting both interviews and allowing for a trial period, both qualitative and quantitative results are collected and analyzed. All different parts are conducted with the same participating families. The next paragraphs elaborate further upon the different parts of this evaluation phase.

##### Interviews Incorporating a Fully Working Proof of Concept

Based upon the evaluation of the paper prototype, a fully working proof of concept is developed using Meteor.js as the underlying cross-platform web architecture. This ensured the proof of concept was compatible with a variety of devices, such as smartphones and tablets, and supported all popular operating systems (eg, iOS and Android). Moreover, the proof-of-concept supported offline caching to prevent any network connectivity issues. Individual semistructured interviews of 60 minutes with young people with 22q11 DS and their parents are conducted at their own homes.

##### Trial Period

After the initial first interviews, the families can use the proof of concept in a trial period lasting 2-4 weeks until the next interview. Families are asked to use the app at least once during this period.

##### Concluding Interviews

During a second individual, semistructured interview session of 45 minutes, families give final feedback. These interviews again take place at their own houses. In these concluding sessions, new insights can be gained after considering the possibility of using the app during a trial period, and feedback from the earlier interview sessions might be confirmed further.

### Participants

During the 2 phases of the study, different groups of participants take part in the study. In the design phase, both medical experts in the field of 22q11 DS and diseases that lead to cognitive impairments and HCI experts are involved. In the evaluation phase, young people with 22q11 DS and their parents are involved.

The medical experts that take part in this research are 2 medical experts that have a proven track record in the field of 22q11 DS and diseases with other cognitive impairments. Besides this, the feedback of 1 female and 2 male HCI experts is gathered in the design phase.

The group of people participating in the evaluation phase are young people with 22q11 DS and their parents and siblings. These young people are required to be between 8 and 23 years of age and need to have taken part in a physical session where the original puzzle was used at least once in their previous treatment. The latter is important, as this research does not want to focus on the contents and workings of the resource and does not want to intervene medically. The parents of the young people did not need to adhere to specific conditions.

### Data Synthesis and Analysis

During the design phase, feedback is gathered about the workings of the low-level paper prototype. Difficulties, possible extensions, and positive feedback are part of this feedback. These findings, in combination with the low-level paper prototype, formed the basis for the development of the proof of concept.

A total of 2 main types of results are acquired in the evaluation phase of the research. First, qualitative results are obtained from both interviews. Besides this, additional quantitative data is obtained from the logs that are collected during the use of the app in the trial period.

Qualitative results are analyzed using thematic analysis [28]. Results are collected and categorized according to the following themes: (1) feedback about the app “as is,” (2) difficulties, (3) comparison between physical and digital tool, (4) extensions, (5) intention, and (6) other.

Quantitative analysis was used to answer questions about the average session length of users and all the different functionalities that were or were not used by families during the trial period.

### Ethics Approval

As this study involves vulnerable participants due to their medical condition and cognitive impairment, ethics approval had to be given by the Ethical Committee for Research at KU Leuven and UZ Leuven to conduct this research. The committee approved the study in March 2022, and it is identified by S-number S66151. Besides approval by the committee, informed consent was obtained from all participants.

## Results

### Overview

This section discusses the most important results from the different parts of the evaluation phase of the research. For the evaluation phase, a total of 8 families were recruited. Table 1 presents more specific details about the different families.

**Table 1:** Overview of the participating families.

ID	Age of the child (years)	Gender of the child	Brothers and sisters, gender, and age (years)
1	9	F^a^	F 6
2	8	M^b^	M 1, M 3, M 5, F 10
3	18	F	M 13, F 16
4	22	M	F 22
5	10	F	F 7
6	18	F	F 13
7	10	F	F 7, M 11, M 13
8	16	M	F 13

^a^F: Female.

^b^M: Male.

### Interviews Incorporating Proof of Concept

#### Overview

**Figure 2:**
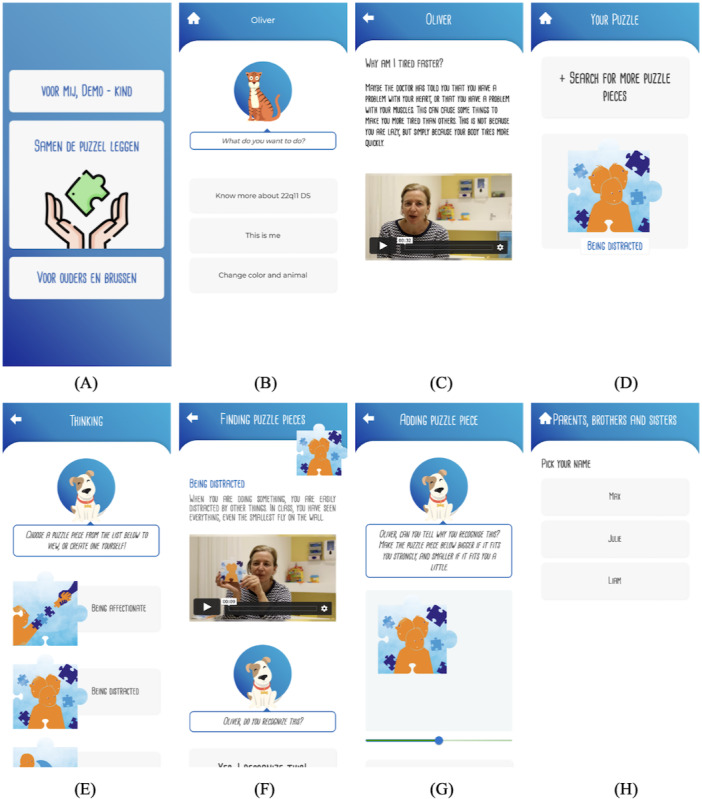
Some of the most important screens in the app: (A) the home screen with links to the 3 main parts, (B) the different functionalities for a child, (C) the way answers to questions are presented to children, (D) the puzzle overview screen, (E) the list of possible puzzle pieces, (F) the way a puzzle piece is presented to a child, (G) the option to make a puzzle piece larger or smaller depending on the level of recognition, and (H) selection screen for parents, brothers, and sisters to navigate to their own part of the app.

During the first semistructured interviews, the first version of the fully working proof of concept is used. An overview of this proof of concept can be found in Figure 2. The app exists out of an onboarding process, functionalities for parents and siblings, functionalities for the person with 22q11 DS, and the possibility to collectively put the puzzle. The functionalities for children exist out of getting answers to frequently asked questions, rating themselves on some skills with stars, and personalizing the app by choosing a color and an avatar. The feedback on the proof of concept is presented according to the categories mentioned earlier.

#### Feedback About the App “as is”

Using the app in general is easy for every child that participates in the study (8/8, 100%). The possibility to choose your own color and avatar is highly appreciated by 87.5% (7/8). Children focus heavily on the visible part of the screen; when presented lists to scroll through, children often choose 1 of the visible parts and seem to minimize scrolling (5/8, 62.5%). If buttons are not visible on the screen immediately, some confusion arises in a few cases (2/8, 25%). Besides this, audiovisual resources show a high impact and get the preference of all the children but one (7/8, 87.5%). Overall, the app receives highly positive feedback from both children and parents, who acknowledge the value such an app can have (8/8, 100%). A striking example of this is one of the younger children (aged 10 years) answering the final question, “Do you have any additional comments you want to add to our conversation?” with, “Will you not forget to let us know where we can find the app so I can use it in the future?” or multiple parents mentioning, “I’m sure this will help a lot of other families.”

#### Difficulties

The main difficulties that arise can be classified into 2 main themes. First, everything text-related should be thoroughly thought about. Difficult words and long sentences cause problems and a loss of engagement during the use of the app, mainly for younger children (5/8, 62.5%). Besides this, as mentioned before, when buttons are not immediately visible on the screen, this can cause confusion as well (2/8, 25%).

#### Comparison Between Physical and Digital Tool

When comparing the physical and digital tool, families sometimes explicitly mention preferring the digital version (4/8, 50%). The burden of using this tool is lower, often because of practical arguments. Using a physical tool simply requires more effort and energy. One of the parents states:

Just having to walk to the closet in the other room and taking the puzzle out is already a burden to use it whereas this is not the case with a mobile application.

Besides, families indicate that the fact that the puzzle is saved creates new opportunities. As everything that is done in the app can be undone, families also indicate they would use functionalities quicker than in the physical case (3/8, 37.5%). For example, only 4 blank puzzle pieces are provided in the physical tool, whereas these are unlimited in the app. This leads to families being less afraid to create their own puzzle pieces. Finally, some of the families indicate they do not think a phone is the appropriate medium to use as a tool for communication support within the whole family because of the simple reason that the screen is too small. Being able to use the app on a tablet or even on a computer could solve this issue (3/8, 37.5%).

#### Extensions

The main extensions that come up are additional information for parents, brothers, and sisters (8/8, 100%), the possibility for brothers and sisters to lay their own puzzle (3/8, 37.5%), and introducing a feedback system for asked questions in the app (3/8, 37.5%).

#### Intention

All children but 1 indicated they were interested in further use of the app during the interview itself (7/8, 87.5%). Parents also say they see the additional benefits. In families where children are already older, they indicate the need for the app is not as high, but they do see the value in a similar app for families with younger children (1/8, 12.5%).

#### Other

During the interviews, the Facebook group of the parent association is mentioned multiple times. However, the subjective and more negatively focused nature of this information leads to a lot of people not wanting to be active in this context. They mention the fact that they would have more trust in an app created by a *trusted* third party like a hospital or government institution (4/8, 50%). Finally, in every family, at the end of the interview, parents emphasized the importance or added value of this kind of research (8/8, 100%).

### Quantitative Results From the Logs Generated During the Trial Period

**Figure 3:**
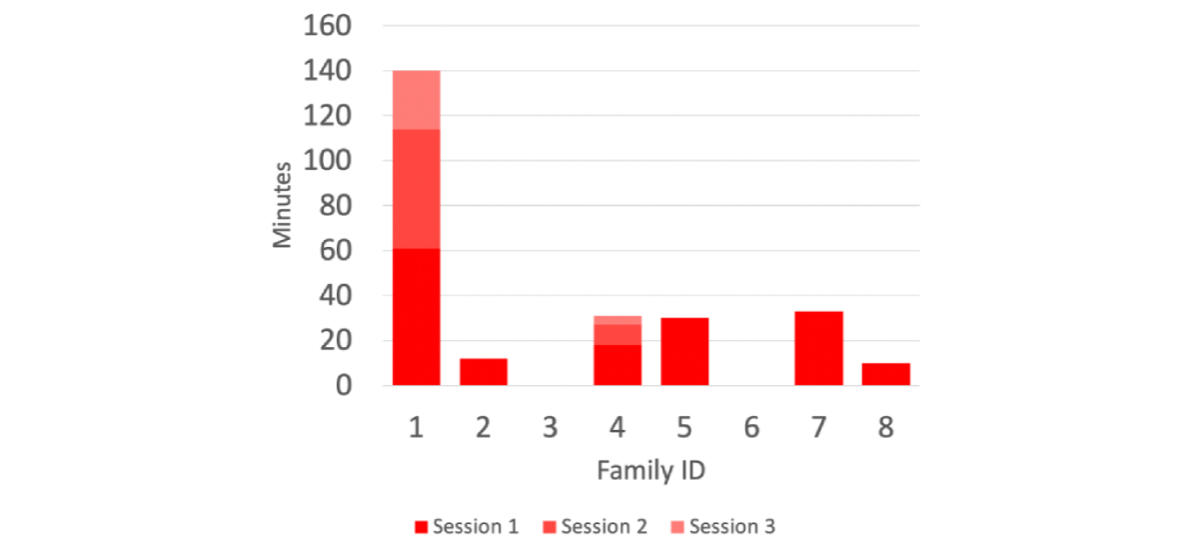
Sessions per family.

While evaluating the usage of the app by looking at the generated logs, a few things became very apparent. A total of 2 families with older children (both 18 years of age) did not use the app during the trial period, even though this was asked at the end of the previous interview and in an email that was sent shortly after the interview. However, in families that did use the app, half of the sessions were 26 minutes or longer, with 2 sessions even lasting 53 and 61 minutes. No technological issues were reported or logged. An overview of the different sessions per family and their duration can be found in Figure 3.

When looking at the functionalities that were used by children and their parents, it can be observed that, overall, the puzzle got a lot of attention.

### Concluding Interview

The families with older children that did not use the app during the trial period indicated they did not have the need to do so. As the syndrome is not an active subject anymore within the family, the need for communication support apps is also lessened. However, they both indicated that if a more urgent situation came up, they would use the app as a tool to support them in their conversations.

In families that did use the app, it was spontaneously mentioned that they had talked about things they had never talked about before. However, one family indicated that right now the puzzle was not yet interactive enough to keep the children’s attention while using it. No additional difficulties came up, except for the fact that one time the child did not understand why the puzzle piece could be made larger or smaller. Children keep preferring the digital puzzle. Finally, one child proposes to extend the app with the possibility of being able to capture pictures themselves to use as images for the puzzle pieces.

Last but not least, it was further confirmed that there is a great need to further involve siblings. Giving them an equally important role in the app is a step forward. There is also a great need for informing siblings, and besides, the ability for every member to put the puzzle together based on their own experiences creates starting points for new, valuable conversations.

## Discussion

### Principal Findings

To summarize our findings, young people with 22q11 DS and their families highly value the developed mHealth app as a supporting tool in communication and for gaining additional information. This confirms the need for these kinds of solutions for families with children with cognitive impairments [15,29-31]. It is highly encouraged that institutions like hospitals or governments take action and start the development of this kind of tool. One should keep in mind that combining powers is a better approach than developing stand-alone apps that all need individual maintenance. This is especially true for apps such as the one discussed in this research, as it shows value for a lot of different target groups.

In general, people with 22q11 DS show few difficulties in using the developed mobile app. This confirms the conclusion from other research that remote interventions with this target group can be successful [21]. It also confirms the earlier presumption that no motorical difficulties would arise, as children with Down syndrome were shown to be able to use mobile apps as well in earlier research [22,23].

Furthermore, while doing interviews with people with 22q11 DS, a couple of things stood out. The fact they are *pleasers* [15] shows when answering the questions in the interviews. It happens regularly when a child indicates they understand something, whereas if asked to perform a certain action, it becomes clear they do not understand this at all. The verbal IQ of these children is often higher than their performance IQ, but this holds the potential risk of overestimating their capacities [32,33].

One of the strengths of this research is that it combines the confirmation of an important need with concrete considerations when developing for people with cognitive disabilities, like people with 22q11 DS. The latter are extensively discussed in the following paragraphs.

### Considerations When Developing Mobile Apps for People With Cognitive Impairments

#### Overview

**Figure 4:**
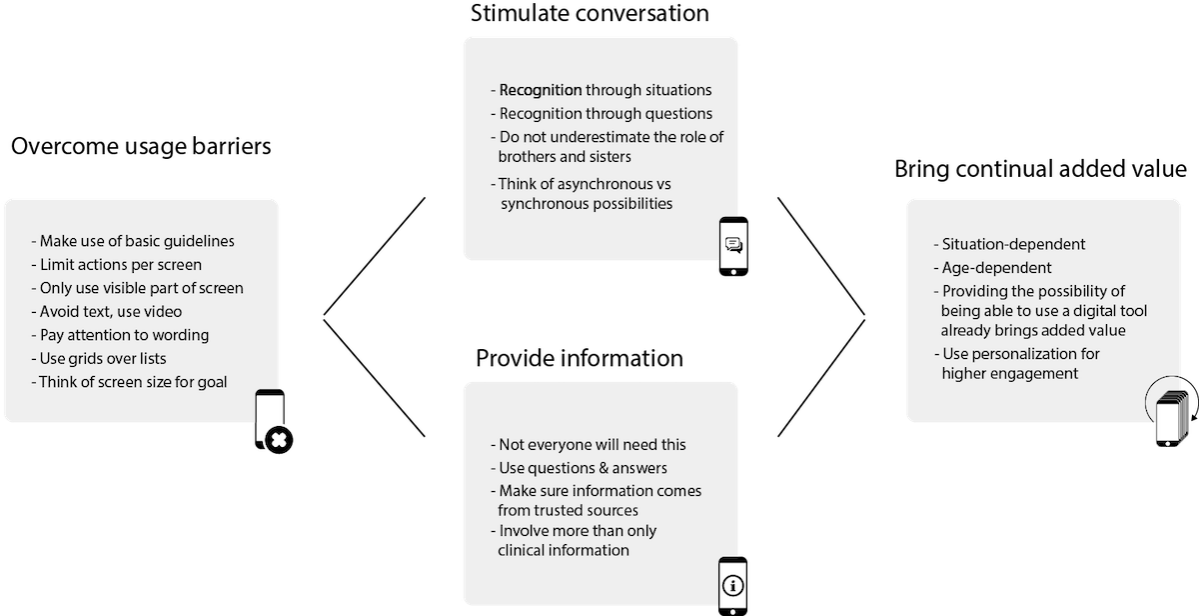
Schematic overview of considerations when developing for people with cognitive impairments in the context of bridging the communication gap.

This research provides insights that should be considered when developing a mobile app for people with cognitive disabilities in the context of bridging the communication gap. These insights can serve as general guidelines. The insights are discussed according to the three-step structure displayed in Figure 4 below: (1) what are the most prominent usage barriers to using mHealth apps, and how can one overcome these? (2) How to achieve desired results, in this case, lowering the communication burden within families and providing information to families? (3) What are the important implications in the context of having continual added value for the target group?

#### Usage Barriers

First and foremost, from this research, it is clear that if the guidelines for developing for the web for people with cognitive impairments are followed when developing mobile apps [34,35], usage barriers are limited. However, one area where it is necessary to be extra cautious is everything related to text. The research shows that using too much text or too difficult words leads to a loss of engagement and interest among young people with 22q11 DS. However, other important design considerations are shown to be important to make sure the mobile app can be easily used. When designing specifically for young people with 22q11 DS, these considerations should be taken into account:

- Limit the actions needed to 1 action per screen.
- Keep all the necessary information directly visible on the screen without scrolling.
- Use audiovisual means wherever possible.
- Avoid long sentences and large collections of text.
- Pay close attention to the words you use; the easier, the better.
- Use grids over lists (earlier research concluded this as well [36]).

Finally, specifically in the context of lowering the communication burden by providing a tool that can be used together with the whole family, some participants indicate that a mobile phone might not be the right medium due to the limited screen size. Families prefer to use a larger screen in this specific situation; for example, using a tablet offers more potential in this area.

#### Achieving Desired Results: Lowering the Communication Burden

With the goal of lowering the communication burden, the concept of *recognition* played a key role. By creating points of recognition using a mobile app, conversation starters are offered to families to talk about more difficult subjects. It was indicated by participants that having these starting points for conversations is in itself enough to lower the communication burden. To maximize the lowering of the communication burden, one can look for various ways to introduce these points of recognition and conversation starters, not only through the existing puzzle pieces, but also, for example, by including testimonials and videos of other people with the same syndrome.

The concept of *recognition* that appears can be found in tools created for other target groups as well. One could argue that reminiscence is a specific kind of recognition. For example, stimulating reminiscence through technology with older adults is found to have a positive effect on communication both in people with and without cognitive impairments [37,38]. Similarly, in this research, using technology to make people think not about situations in the past but about situations in the present appears to be an important element that can stimulate conversations.

Besides, in this research, the very important role played by the brothers and sisters of the child with 22q11 DS appeared. It is important to involve these siblings heavily, as they both have their own questions and challenges but are also some of the people who know the child with 22q11 DS best [39-41]. This research implements some recommendations for practice, like encouraging siblings’ curiosity about the mindset of their brother or sister with a disorder and inviting the sibling to discuss issues regarding feeling normal and feeling different [41]. It also confirms the fact that feedback from siblings is highly valuable during research itself [41].

Finally, in the field of communication, it should be noted that families tend to use a mobile app on a more individual basis than a physical tool. Conversations occur not only synchronously while using the app together but also asynchronously. For example, children find things they think are interesting while using the app and afterwards tell their parents about them and start new conversations.

#### Achieving Desired Results: Providing Information

For the goal of providing information to people with 22q11 DS and their caregivers, one should be conscious of the fact that not all children with 22q11 DS have the need to have a deeper understanding of why things are the way they are. This is largely attributed to having cognitive impairments [42,43]. However, when presenting information to them, using video and information at their level of thinking are crucial aspects to being successful in this goal.

When presenting clinical information to parents, a question-answer system split up into different categories seems like a potential way to go. What they think is especially important is a trusted third party that provides the information. Therefore, it should be encouraged that official institutions with knowledge about 22q11 DS are the creators of these kinds of apps and provide the necessary maintenance. An important consideration for practice also involves the fact that parents are not only interested in clinical information. They are as interested in the practical consequences of having to manage a child with 22q11 DS regarding taxes, institutions, support organizations, and other related topics.

#### Bringing Continual Added Value

Having continual added value has been shown to be a complex topic in this research. From the feedback and effective usage of the app, it becomes clear that not every family needs regular conversations about the syndrome. What families need are tools that can support them at the moments they need them. In families with younger children, this will be a more permanent situation, whereas in families with older children, only at the most urgent points in time will an app be used.

However, just having the option of using a digital tool like the one in this research is in itself already a way to bring continual added value. Families indicate they would more quickly use a digital tool than a physical tool, solely because of the lower practical burdens of using it.

Last but not least, in order to boost engagement, personalization has been shown both in previous research [44] and in this study to be an important aspect for the success of an app.

### Limitations

A few important limitations need to be pointed out in this research. First, although 8 different families with children with 22q11 DS took part in the research, the quantitative analysis of the logs with more participants could lead to even more valuable insights. Nonetheless, our participants provide important perspectives that enhance our understanding of their situation. Involving the families provided additional information and firsthand knowledge gained through years of experience and interaction with health professionals. We employed a mixed methods approach, combining qualitative methods like in-depth interviews with quantitative data after real-world usage, enabling a comprehensive exploration of participants’ experiences and needs. However, the trial period in this study can be perceived as relatively short. On the other hand, the trial period did provide us with valuable additional insights that would not have been collected with interviews alone. This study serves as an exploratory investigation, laying the groundwork for future research and informing studies with larger sample sizes, leading to a gradual expansion of knowledge in the field.

### Conclusions

In this research, an iterative, user-centered design process is carried out. Based on the design phase, a fully working proof of concept of a mobile app is developed with the goal of bridging the communication gap between people with 22q11 DS and their caregivers. This proof of concept is evaluated during the evaluation phase. The need for these kinds of apps is confirmed. All participating families express their gratitude and mention the added value for other families. Therefore, it is highly encouraged for institutions to act and develop an app to be used in practice. Furthermore, considerations when developing for people with 22q11 DS, or more broadly, people with cognitive impairments, are proposed. First, one should keep design principles in mind to overcome usage barriers. Next, recognition is a key concept when stimulating conversations through mobile apps. Third, information should be provided by a trusted source, and not only clinical information brings added value. Finally, having the possibility of using a digital tool that can be personalized brings continual added value.

## Abbreviations

22q11 DS: 22q11 deletion syndrome
HCI: human computer interaction
NDD-CNV: neurodevelopmental disorder due to a copy number variant
